# Physicochemical properties and effect of bioceramic root canal filling for primary teeth on osteoblast biology

**DOI:** 10.1590/1678-7757-2020-0870

**Published:** 2021-05-03

**Authors:** Victor Manuel OCHOA RODRÍGUEZ, Mario TANOMARU-FILHO, Elisandra Márcia RODRIGUES, Eduarda de Oliveira BUGANÇA, Juliane Maria GUERREIRO-TANOMARU, Gisele FARIA

**Affiliations:** 1 Universidade Estadual Paulista “Julio de Mesquita Fillho” Faculdade de Odontologia de Araraquara Departamento de Odontologia Restauradora AraraquaraSP Brasil Universidade Estadual Paulista “Julio de Mesquita Fillho” (UNESP) - Faculdade de Odontologia de Araraquara - Departamento de Odontologia Restauradora - Araraquara - SP - Brasil.

**Keywords:** Cytotoxicity, Endodontics, Primary teeth, Root Canal Filling Materials

## Abstract

Bio-C Pulpecto (Bio-CP) was recently developed as the first bioceramic root filling material for primary teeth.

**Objective:**

To evaluate the physicochemical properties of radiopacity, setting time, pH, cytocompatibility and potential of Bio-CP to induce mineralisation, compared with (1) Calen thickened with zinc oxide (Calen-ZO), and (2) zinc oxide and eugenol (ZOE).

**Methodology:**

Physicochemical properties were evaluated according to ISO 6876. Saos-2 (human osteoblast-like cell line) exposed to extracts of the materials were subjected to assays of methyl thiazolyl tetrazolium, neutral red, alkaline phosphatase (ALP) activity and mineralised nodule production. The results were analysed using one-way or two-way ANOVA and Tukey’s or Bonferroni’s post-tests (α=0.05).

**Results:**

All the materials showed radiopacity higher than 3 mm Al. Bio-CP had lower pH than Calen-ZO, but higher pH than ZOE. Calen-ZO and Bio-CP did not set. The setting time for ZOE was 110 min. The cytocompatibility order was Calen-ZO > Bio-CP > ZOE (1:2, 1:4 dilutions) and Calen-ZO > Bio-CP = ZOE (1:12, 1:24 dilutions) and Calen-ZO = Bio-CP > ZOE (1:32 dilution). Bio-CP induced greater ALP activity at 7 days, and greater mineralised nodule production, compared to Calen-ZO (p<0.05). Conclusions Bio-CP showed adequate physicochemical properties, cytocompatibility and potential to induce mineralisation.

## Introduction

An ideal root canal filling for primary teeth should not hinder the eruption of permanent successor teeth, but rather, should be resorbed as the deciduous tooth roots are physiologically resorbed, and should also resorb readily if pressed beyond the apex, be easily removed if necessary, be radiopaque and not discolour the tooth.^1^

Zinc oxide and eugenol (ZOE) are a combination that has been used as a root canal filling material in primary dentition for a long time.^2,3^ However, ZOE is genotoxic and cytotoxic,^4^ and cannot be completely phagocytised, leaving particles in periapical tissues when extravasated beyond the apex, and after physiological root resorption of the deciduous teeth.^2^

Calen (S.S. White, Rio de Janeiro, RJ, Brazil) is a commercial calcium hydroxide-based paste with a viscous vehicle (i.e., polyethylene glycol 400), and has suitable biological properties.^5^ Calen thickened with zinc oxide (Calen-ZO) has been used as a root canal filling material in the primary dentition.^6-8^ The addition of zinc oxide (ZO) reduces the phagocytosis of Calen, thus allowing Calen to accompany the physiological resorption of primary tooth roots, and improve its physicochemical properties.^7,9^ Calen-ZO induces good tissue response,^7,10^ has antimicrobial activity^6^, and is more biocompatible than ZOE.^7,8^

New endodontic cements have been developed, especially highlighting the advance in improved bioceramics. The main characteristics of bioceramic materials for endodontic use are their alkaline pH, shrink-free property, chemical stability within the biological environment,^11,12^ biocompatibility and bioactivity.^13,14^Bio-C Pulpecto (Bio-CP) (Angelus, Basil, Londrina, Paraná, Brazil) is the first bioceramic root filling material for primary teeth. It is composed of ester glycol salicylate, titanium oxide, calcium tungstate, silicon dioxide, toluene sulphonamide, and calcium silicate (Material Safety Data Sheet information, product in development, file annexed). According to its manufacturer, it presents high alkalinity (pH 12.7), high radiopacity (9 mm of aluminium scale), complies with ISO 6876,^15^ and is resorbable, thus allowing simultaneous physiological resorption of the root and the material (Bio-C Pulpecto, Angelus, Basil, Londrina, Paraná, Brazil). A recent study showed that the Bio-CP is biocompatible and induced biomineralization in the subcutaneous tissue of rats, in a manner similar to MTA.^16^ In addition, a root filling material for primary teeth developed by Angelus, with similar composition of Bio-CP, but with the name not informed, showed good biocompatibility in the subcutaneous tissue of rats, low cytotoxicity in human gingival fibroblast, and satisfactory behaviour regarding the physicochemical properties studied.^8^ However, the scientific literature about Bio-CP is still scarce.

Thus, the aim of this study was to evaluate (1) the physicochemical properties of radiopacity, setting time and pH, and (2) the biological properties of cytocompatibility and potential to induce mineralisation of Bio-CP, compared with Calen-ZO and ZOE, *in vitro* assays. The null hypothesis was that there would be no difference in the physicochemical and biological properties among the materials.

## Methodology

**Figure 1:**
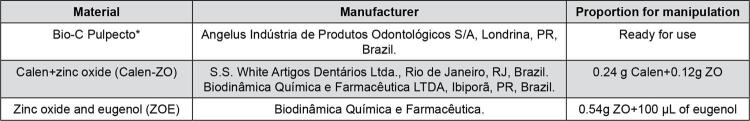
Root canal filling material, manufacturer and proportion of use. * Product under development, batch no. 190118

Root filling materials, manufacturers and proportions for manipulation are shown in Figure 1.

### Radiopacity evaluation

Eight specimens of each material, measuring 10 mm diameter and 1 mm high were prepared according to ISO 6876:2012^15^ (Figure 1). The specimens were stored at 37°C and 95% humidity for 3 times the setting time for ZOE, until achieving maximum hardening for Bio-CP and Calen-ZO. Afterwards, they were placed on top of a KODAK CMOS digital sensor (6100, Kodak Co.), next to an aluminium step-wedge (98.5% of Al, 8 steps with 2 mm increments for each step) for radiographic exposure. Focus 50540 x-ray unit (Instrumentarium Dental; Tuusula, Finland) was used, and the parameters were set at 65 kVp, 7 mA, exposure time of 0.16 seconds and a 320 mm source-to-object distance.^17^The digital images obtained were evaluated using methodology developed by Ochoa-Rodriguez, et al.^17^ (2019).

### pH analysis

Ten polyethylene tubes 10-mm long with 1 mm internal diameter were filled with each material. Each specimen was immersed in 10 mL of deionised water, inside of a plastic flask sealed individually with a lid, and then stored at 37°C. The experimental time intervals were 1, 3, 7, 14 and 28 days. The pH of the solution was assessed with a previously calibrated digital pH meter (Digimed; São Paulo, SP, Brazil) at each time interval. The control group consisted of deionised water with no immersed material.

### Setting time

Calen-ZO and ZOE were inserted into ring-shaped metal moulds 1 mm wide and 10 mm diameter (n=6), kept at 37°C and 95% humidity. A plaster mould with the same dimensions was prepared for Bio-CP. The plaster mould was previously submerged in deionised water for 24 hours to provide the bioceramic material with moisture. The setting time was assessed using a Gillmore needle with a mass of 100 g ±0.5 and diameter of 2.0 mm ±0.1, according to ISO 6876:2012.^15^ The setting time of the specimens was established as the time elapsing from initial mixing of Calen-ZO and syringe application of Bio-CP up to the maximum hardening of the specimens. At this point, the indentation marks made by the Gillmore needle were substantially reduced, but not eliminated. This is because the pastes had not set. Comparatively, the setting time for ZOE was considered as the time elapsing from when it started to be mixed until such time as the Gillmore needle failed to leave marks on the surface of the specimens.

### Cell culture and preparation of root filling materials

Saos-2 (human osteoblast-like cell) immortalized cells line were grown in flasks containing Dulbecco’s Modified Eagle Medium (DMEM; Sigma-Aldrich, St. Louis, MO, USA) supplemented with 10% fetal bovine serum (FBS, Gibco Life Technologies, Grand Island, NY, USA), penicillin (100 IU / mL), and streptomycin (100 μg / mL), in an atmosphere with 5% CO2 and 95% humidity at 37°C.

After handling of the specimens, 100 mg of each material (Figure 1) was placed in a 1.5 mL microtube (Eppendorf, Hamburg, Germany), to which 1.2 mL of DMEM was added, followed by placement of the microtubes in an oven at 37°C for 24 hours.^18^ Afterwards, the supernatant was transferred to new microtubes centrifuged for 10 min at 20,800 g (5430, Eppendorf AG, Hamburg, Germany) to decant any particles of material left in the supernatant. The supernatant was transferred to a new tube and was considered as the “stock solution/extract”. It was then diluted and placed in contact with Saos-2 cells.^19^

### Cell viability analysis by methyl thiazolyl tetrazolium (MTT) and neutral red (NR) assays

Saos-2 cells were cultured at a density of 1x10^5^ cells/mL in a 96-well plate containing DMEM with 10% SFB for 24 hours to adhere to the plates. Thereafter, the cells were exposed to the cement extracts at the following dilutions in DMEM (v: v) 1:2; 1:4; 1:12; 1:24; 1:32. Cells exposed to DMEM were considered as the control group.

After 24 hours of cell contact with the material extracts and the control, 100 μL of 5 mg / mL MTT solution (Sigma-Aldrich) was added to each well, followed by incubation at 37°C, 95% humidity and 5% CO_2_ for 3 hours. The colorimetric product was solubilised in 100 µl of 0.04 N acidified isopropanol (Sigma-Aldrich). A spectrophotometer (Elx800; Bio-Tek Instruments, Winooski, VT, USA) at 570 nm was used to measure the optical densities of the solutions. Absorbance readings were normalised to readings of cells exposed to DMEM, and represented the succinate dehydrogenase activity (cellular metabolism).

The NR assay was performed by applying 100 μL DMEM containing NR at 50µg / mL (Sigma-Aldrich) after 24 hours of cell contact with the materials and control extracts. The cells were incubated at 37°C, 95% humidity and 5% CO_2_ for 3 hours, the contents of the wells were removed, and the colorimetric product was solubilised with 100 μL of a solution containing 50% ethanol and 1% acetic acid (Sigma-Aldrich). A spectrophotometer (Elx800; Bio-Tek Instruments, Winooski, VT, USA) at 570 nm was used to measure the optical densities of the solutions. Absorbance readings were normalised to readings of cells exposed to DMEM, and represented the ability to incorporate the dye into viable cell lysosomes.

### Alkaline phosphatase (ALP) activity

Saos-2 (5x10^4^ cells / mL) were cultivated in 96-well plates and exposed to the control (DMEM) and the extract of the materials at 1:24 dilution. This dilution was selected after observing the results of MTT assays performed after exposure of cells to cement extracts for 1, 3 and 7 days (data not shown). The 1:24 dilution was the highest extract concentration without cytotoxic effects. This is an essential consideration, since dead cells do not show ALP activity. ALP activity was evaluated at periods of 1, 3 and 7 days, using the commercial kit (Labtest, Lagoa Santa, MG, Brazil). Extracts of the materials were renewed every two days. Cells were washed with 200 μL saline phosphate buffer (PBS) after each experimental period, and a 200 μL sodium sulphate solution (1% in distilled water, Sigma-Aldrich) was added to each well. Then the samples were left to stand for 30 minutes at room temperature. Each sample (5 μL) in the sodium sulphate solution was transferred to a microtube (Eppendorf) containing a substrate and a buffer enzyme. Absorbance was measured using a spectrophotometer at 590 nm. Data were expressed as normalised ALP activity with total protein content, detected using the Total Protein Kit (Labtest) at the respective experimental periods. MTT assays were performed together with the ALP activity assay to follow up cell viability.

### Alizarin Red Staining (ARS)

Saos-2 cells were cultivated (1x10^4^ cells / mL) in 12-well culture plates with DMEM, supplemented with 50 µg / mL L-ascorbic acid (Sigma-Aldrich) and 10 mM β glycerophosphate (Sigma-Aldrich). The cells were exposed to material extracts for 21 days at the same dilution used for the ALP activity assay. Material extracts were renewed every two days. Then the cells were fixed with 4% paraformaldehyde (Sigma) and stained with 2% ARS (pH 4.1). The cells were incubated at room temperature for 20 minutes, and the dye was aspirated. The wells were washed 4 times with 1 mL of distilled water for 5 minutes. The water was removed and mineralisation was quantified by dissolving the nodules with 1 mL of a 10% cetylpyridinium chloride solution (Sigma Aldrich) added to each well, after which the plate was incubated for 15 minutes under stirring at room temperature. Three 100 μL aliquots of the suspension from each well were transferred to a 96-well plate and read with a 562 nm wavelength filter on a spectrometer (Elx800; Bio-Tek Instruments).

### Statistical analyses

The results were shown as mean and standard deviation in at least two independent experiments performed from triplicate to sextuplicate. The results were analysed by one-way analysis of variance (ANOVA) and the Tukey’s post-test, or two-way ANOVA and Bonferroni’s post-test (α=0.05), using GraphPad Prism statistical software (GraphPad Software; San Diego, CA, USA).

## Results

### Physicochemical properties

According to Table 1, all the materials showed radiopacity higher than 3 mm Al, in agreement with ISO 6876:2012.^15^ There was no difference between Bio-CP and Calen-ZO (p>0.05), and ZOE showed higher radiopacity than the other materials (p<0.05). The ZOE setting time was 110 minutes. Calen-ZO and Bio-CP were considered to “set” (maximum hardening) at 192 hours and 240 hours, respectively. Although Calen-ZO and Bio-CP pastes had alkaline pH, Calen-ZO had a higher pH than Bio-CP (p<0.05). ZOE had a neutral pH (Table 2).

**Table 1:** Different letters in the lines indicate statistically significant differences among the materials (P<0.05) *The material did not set after the elapsed “setting” time. ** Partial areas of the material set at the evaluated time

	Bio-CP	Calen-ZO	ZOE
Radiopacity	3.50 (0.15)^a^	3.51 (0.12)^a^	9.25 (0.31)^b^
Setting time	240.7 (0.1)^a*^	192.0 (0.2)^b**^	1.822 (0.01)^c^

Different letters in the lines indicate statistically significant differences among the materials (P<0.05).

*The material did not set after the elapsed “setting” time. ** Partial areas of the material set at the evaluated time.

**Table 2:** Different letters in the lines indicate statistically significant differences among materials (P<0.05)

	Bio-CP	Calen-ZO	ZOE	Control
1 day	10.07 (0.22)^b^	11.32 (0.16)^c^	7.30 (0.16)^d^	7.04 (0.08)^a^
3 days	9.92 (0.30)^b^	10.84 (0.22)^c^	7.18 (0.16)^a^	6.94 (0.07)^a^
7 days	8.57 (0.70)^b^	10.34 (0.38)^c^	7.03 (0.17)^a^	6.94 (0.08)^a^
14 days	9.65 (0.29)^b^	10.01 (0.68)^b^	6.87 (0.29)^a^	6.50 (0.25)^a^
21 days	8.70 (0.45)^b^	9.70 (0.63)^c^	6.99 (0.15)^a^	6.64 (0.16)^a^
28 days	8.08 (0.73)^b^	9.65 (0.73)^c^	6.52 (0.07)^d^	5.79 (0.14)^a^

Different letters in the lines indicate statistically significant differences among the materials (P<0.05).

### Cell viability

**Figure 2:**
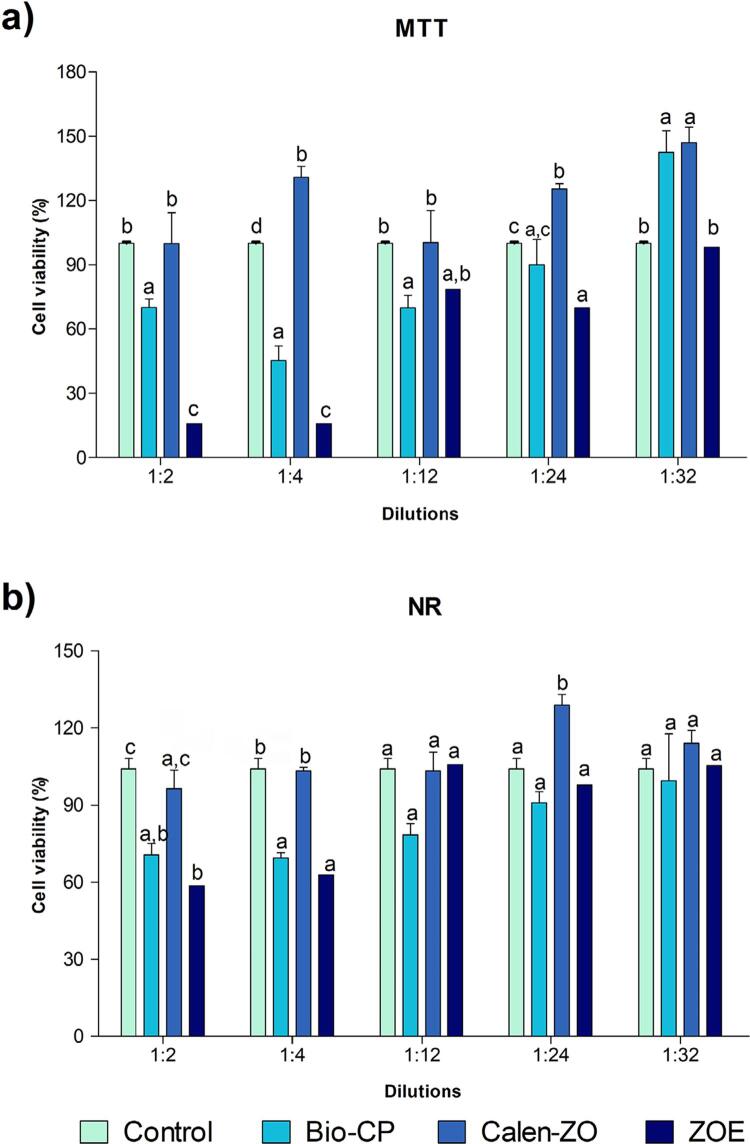
Saos-2 viability evaluated by (a) methyl-thiazol-tetrazolium (MTT) and (b) neutral red (NR) assays, after 24 hours of exposure to Bio-CP, Calen-ZO, ZOE and control (culture medium). Bio-CP=Bio C Pulpecto; Calen-ZO=Calen mixed with zinc oxide; ZOE=Zinc oxide and eugenol. Different letters in each dilution indicate statistically significant differences among the materials (p<0.05)

As shown in Figure 2, the cell viability order at lower dilutions (1:2 and 1:4) was Calen-ZO > Bio-CP > ZOE for MTT and Calen-ZO > Bio-CP = ZOE for NR. As for the intermediate dilutions (1:12 and 1:24), the Calen group had the highest cell viability (p<0.05) in the MTT assay, whereas the Bio-CP and ZOE groups had similar cell viability (p>0.05). As for the NR assay, there was no difference between the materials and the control (p>0.05), except Calen-ZO at 1:24 dilution, that had higher cell viability than the other groups (p<0.05). At the highest dilution (1:32), there was no difference between Calen-ZO and Bio-CP (p>0.05) in the MTT assay, and both groups had higher cell viability than ZOE. In the NR assay, there was no difference among the materials evaluated at 1:32 dilution (p>0.05).

### Potential to induce mineralisation

**Figure 3:**
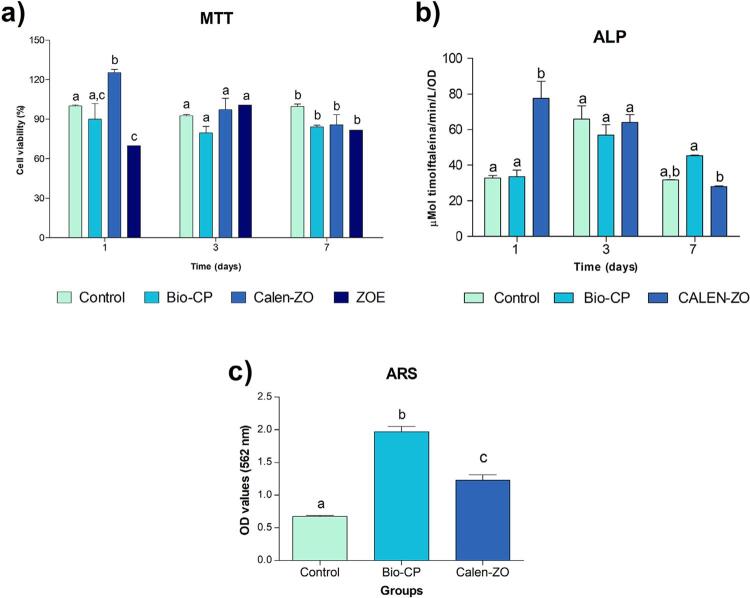
Effect of root canal filling materials (1:24 dilution) of primary teeth on the biology of Saos-2. MTT assay (a) and alkaline phosphatase (ALP) activity (b) were performed 1, 3 and 7 days after exposure of Soas-2 to extracts of materials and serum-free culture medium (control), whereas alizarin red stain (ARS ) assay (c) was evaluated after 21 days of cell exposure to material extracts and osteogenic culture medium (control). Bio-CP=Bio C Pulpecto; Calen-ZO=Calen mixed with zinc oxide and eugenol; ZOE=Zinc oxide and eugenol. Different letters indicate statistically significant differences among the materials (p<0.05)

As shown in Figure 3, on the first and third day of Saos-2 exposure to the materials, all groups had similar (p>0.05) or greater cell viability than the control group (p<0.05), except for ZOE, which promoted lower cell viability than the control group (p<0.05). On the seventh day, there was no difference in the viability of the cells exposed to all the materials and the control (p>0.05). On the first day of cell culture, Calen-ZO induced higher ALP activity than Bio-CP and the control (p<0.05), whereas Bio-CP promoted ALP activity similar to that of the control (p>0.05). On the third day, there was no difference among the groups (p>0.05). On the seventh day, Bio-CP induced higher ALP activity than Calen-ZO (p<0.05), whereas Calen-ZO was no different from the control (p>0.05). In 21 days of cell exposure to the materials, Bio-CP and Calen-ZO induced higher mineralised nodule production than the control (p<0.05), although that of Bio-CP was higher than that of Calen-ZO (p<0.05).

## Discussion

In the present study, a comparison was made of the biological and physicochemical properties of Bio-CP, Calen-ZO and ZOE. Bio-CP exhibited adequate physicochemical properties, had cytocompatibility and revealed potential to induce mineralisation, when compared to the other evaluated root canal filling materials for primary teeth.

Radiopacity is an essential property for endodontic materials, because it allows endodontic filling material to be viewed by radiographic examination to evaluate the obturation quality,^12^ and make a clear distinction among the materials and the surrounding anatomical structures.^20^ ISO 6876:2012^15^ establishes that the root canal sealers must have at least 3 mm Al. In the present study, all the materials evaluated showed higher radiopacity than 3 mm Al. However, the radiopacity value of Bio-CP (3.50 mm Al) was less than that stated by the manufacturer (9 mm AL). On the other hand, it agrees with a previous study that showed that an experimental MTA-based material, with similar composition of Bio-CP, had radiopacity of 3.28.^8^ The greater radiopacity of ZOE (9.25 mm Al) than that of the other materials (3.5 mm Al) is attributed to its zinc oxide used as a radiopacifier.^21^

An alkaline medium not only neutralises lactic acid from osteoclasts, but also prevents the mineral components of dentine from dissolving. In addition, it promotes antimicrobial activity, and may activate alkaline phosphatases, which play an important role in hard-tissue formation.^22^ Both Bio-CP and Calen-ZO had alkaline pH; however, Calen-ZO had a higher alkaline pH compared with Bio-CP over the evaluated periods. Calcium hydroxide dissociates into calcium and hydroxyl ions, which raises the pH of the medium. Bioceramic materials form calcium hydroxide when they undergo a hydration reaction.^12^The lower pH of Bio-C compared to Calen-ZO is probably due to the lower amount of calcium hydroxide released into the medium by Bio-CP.

ZOE had a setting time of 110 minutes. The hardening time of Calen-ZO was 192 hours and that of Bio-CP was 240 hours; neither material set. The fact that Bio-CP does not set is not a disadvantage, since this phenomenon is observed in materials considered suitable for filling root canals of primary teeth.^8,9,23^ Because Bio-CP was developed as a root filling material for primary teeth, is desirable that the material can be reabsorbed to accompany the physiological resorption of deciduous teeth roots. It can be hypothesized that the absence of set, along with the composition and solubility of the material, can allow simultaneous physiological resorption of the root and the material.

Saos-2 cells were used because they provide a suitable model to evaluate cytocompatibility and the potential to induce mineralisation of the materials.^17^ MTT and NR assays were used to assess the cytocompatibility of the materials. MTT is based on the ability of viable cells to metabolise the MTT salt. NR assesses the ability of viable cells to incorporate dye into lysosomes.^17^ Studies that evaluate different cell parameters make the cytocompatibility results more reliable^24^ ALP activity and mineral nodule production assays were performed to evaluate the potential of materials to induce mineralisation. ALP activity assay is a biomarker of the enzyme alkaline phosphatase produced by osteoblasts in their early maturation process,^25^ and ARS identifies calcium deposits in cell culture.^17^

The cytocompatibility and the potential to induce mineralisation of bioceramic endodontic materials for permanent teeth has been studied in different cell types. EndoSequence BC sealer (Brasseler USA, Savannah, GA, USA) showed low cytotoxicity in L929 fibroblasts,^26^ human gingival fibroblasts^27^and murine osteoblast precursor cells (IDG- SW3).^13^ Moreover this material promoted osteoblastic differentiation,^13^ showed anti-inflammatory effects and induced mineralisation in osteoblast precursor cells (MC3T3-E1).^14^ Another study comparing mineral trioxide aggregate – MTA (ProRoot; Dentsply Tulsa Dental, Tulsa, OK, USA) and iRoot SP (Innovative BioCreamix, Vancouver, Canada) showed that neither material was cytotoxic in human tooth stem cells.^28^

Bio-CP cytocompatibility was lower compared to Calen-ZO, but higher compared to ZOE. Pilownic, et al.^8^ (2017) showed no statistical difference in the cytotoxicity of Calen-ZO and the experimental MTA-based material with a similar composition of Bio-CP. This difference in result can be explained by the type of cells and also by the Calen-ZO proportion used. While in the present study Saos-2 cells (osteoblast-like cells), and Calen-ZO in a 2: 1 proportion were used, Pilownic, et al. ^8^ (2017) used human gingival fibroblasts and Calen-ZO in a 1: 1 proportion. It is important to emphasize that it is not clear if the manufacturer modified the concentrations of the components in Bio-CP, because at the time of the Pilownic, et al.^8^ (2017) study, the material did not have a name yet and, unfortunately, the manufacturer did not disclose the concentrations.

Bio-CP induced lower ALP activity in 1 day, but greater ALP activity in 7 days, than Calen-ZO. In addition, Bio-CP induced greater mineralised nodule production than Calen-ZO. Calen-ZO induced higher calcium deposits than the control group. A recent study showed that Bio-CP was biocompatible and induced biomineralization similar to MTA in the subcutaneous tissue of rats.^16^ Regarding Calen-ZO, the results of the present study are in line with previous studies performed on dogs’ teeth and the subcutaneous tissue of mice, which showed that Calen-Zo was more biocompatible than ZOE.^7,10^ ZOE was not evaluated in either mineralisation assay conducted in the present study, because it has no bioactivity.^29^The high mineralisation potential of calcium-silicate-based bioceramic materials is attributed to their hydration, that is, a chemical reaction with tissue fluids yields hydrated silicate gel (C-S-H) and calcium hydroxide.^30^ These compounds (C-S-H and calcium hydroxide) each have a mineralisation pathway. Calcium hydroxide induces the formation of calcite crystals that induce the formation of calcified areas. Calcite crystals are formed from the dissociation of calcium ions from calcium hydroxide, and carbon dioxide ions from tissues.^31^ On the other hand, C-S-H induces hydroxyapatite deposition; this reaction is promoted by the hydration of tissue fluids. In bioceramic materials, calcium hydroxide reacts with the phosphate ions in tissue fluids to deposit calcium phosphate.^4,30^

The results obtained for cytocompatibility and mineralization induction should be confirmed by further *in vivo* studies. In addition, to complement the research on Bio-CP, studies should be carried out to evaluate its antibiofilm activity and the capacity to be reabsorbed to accompany the physiological resorption of deciduous teeth roots.

It was concluded that Bio-CP did not set, presented adequate radiopacity and alkaline pH, was cytocompatible and had the potential to induce mineralisation. Considering its good physicochemical and biological properties, Bio-CP has the potential to become an adequate material for the root canal filling of primary teeth.
